# Richmond Academy of Medicine and Surgery

**Published:** 1896-12

**Authors:** J. W. Long

**Affiliations:** Richmond, Va., Professor of Diseases of Women, Medical College of Virginia


For the Texas Medical Journal.

### RICHMOND ACADEMY OF MEDICINE AND SURGERY.

### Cystoscopy and Ureteroscopy with Exhibit of Instruments and Report of Cases.

BY J. W. LONG, M. D., OF RICHMOND, VA.,
Professor of Diseases of Women, Medical College of Virginia.

ONLY RECENTLY has the bladder received the attention it deserves from gynecologists. Up to the last few years it was difficult to explore that viscus to determine the condition of its inner surface, and more difficult to explore the ureters. Prior to the invention of the instruments I am about to exhibit, we were obliged to depend upon the dilatation of the urethra by a very crude method; indeed, stretching by the fingers, which often brought on permanent incontinence, is within the recollection of most of us. To explore the bladder was almost impossible, because of callapse of its walls, and, as said before, exploration and treatment of the ureters was attended with even more difficulty. For many years distension of the bladder with water, and fishing around for the mouths of the ureters, were our only resources. That until Kelly accidently struck upon the method to be described could we satisfactorily treat cystic and ureteral troubles. The method depends on, first, distension of the bladder with air, which, a few years ago, was thought to be exceedingly dangerous; and second, instruments whereby we may have a direct view of the inside of the bladder and the mouths of the ureters.

The instruments I show you are, first, sixteen dilators, graded according to their measurement by the metric system. Numbers 10 to 14 are those usually employed. It is surprising to note the amount of treating the female urethra can stand.

The second instrument is the calibrator, so called because, originally, it was intended to measure the urethra. It is cone-shaped and graduated, and is now used in the place of the original dilators.

At an early stage in the work it was found that the external meatus was the point of greatest resistance in dilating the urethra.

The third instrument is the cystoscope (made in various sizes), consisting of a straight tube flanged at one extremity, and having, projecting at an angle from near the same extremity, a handle. Fitting in this is an obturator. After the urethra is dilated the cystocope is forced in by a gentle rotary movement, and when the obturator is withdrawn air rushes in, distending the bladder. I have suggested to Dr. Kelly that the cystocope might, to advantage, be made shorter and with a more flaring flange.

The position the patient was made to assume at first was the dorsal, with hips much elevated. I have devised a pelvis elevator for this purpose. The bladder is first catheterized, the urethra dilated, the cystoscope introduced, and the residual urine drawn off by a suction apparatus, but it is almost a matter of impossibility to keep the bladder entirely clear of urine. A better posture is the knee-chest, because in it the residual urine gravitates from the floor of the bladder to the most dependent part, and the suction apparatus may be dispensed with. After exploring the entire inner surface of the bladder, whieh can be seen perfectly, it is easy to find the mouths of the ureters. Any kind of clear, reflected light can be used with a head mirror.

Through the cystoscope can be introduced the ureteral searcher, which is a small sound with the handle bent at an obtuse angle. At first this is a difficult procedure, but practice makes it easy. I tried for eighteen months to catherize the ureters, and had about decided that it was one of the things I could not do, when I suddenly hit upon it, and can now introduce the ureteral catheter in a few seconds.

The original ureteral catheter was devised by Simon. Pawlik modified it, and Kelley perfected the Pawlik instrument.

With a delicate pair of mouse-tooth forceps, it is not at all difficult to pick a piece of cotton, stone, etc., from the bladder; or, with an applicator, to make an application into its walls.

Whale-bone bougies are employed, first, to determine whether or not the ureters are potulous; second, to protect them during hysterectomy.

The vesical balloon, devised by Dr. Clark, consists of a fine rubber bag, with a stem of rubber tubing. It is used for the treatment of chronic vesical catarrh where there are marked changes in the bladder walls, and which are not amenable to treatment by ordinary applications. He employs it covered with a 10 per cent gelatole of ichthyol, melting at the temperature of the body. The balloon is buttered with the gelatole, rolled up, grasped in a pair of special forceps, introduced through the cystoscope, and distended with air; the medicine thereby reaching every part of the inner surface of the bladder. To prevent injury or rupture of the bladder, it is important to learn first how far the balloon should be distended.

By means of the cystoscope it has been discovered that many of the so-called cases of cystitis, especially in young women, are not general inflammations of the vesical mucosa, but hyperdermia, or, at most, limited inflammations; and many of them are easily and rapidly cured by mild solutions of nitrate of silver, or any other astringent, applied through the cystoscope.

I shall cite only one or two cases, to demonstrate its efficiency.

Case 1 was that of a young woman, married, who for years had had cystic trouble. Under cocaine anesthesia, the cystoscope being introduced and the area of inflammation determined, three or four applications of nitrate of silver sufficed to cure.

Case 2.—A young woman whose tubes and ovaries had been removed two years before I saw her, for probably avaro-salpingitis. After the operation ventral hernia occurred, and she came for treatment. Inquiry revealed more suffering from the bladder than from the hernia, and brought to light the fact that for thirteen years she had been the victim of cystic trouble. There was very little pus in the urine, but a slow, irritating, aggravating cystitis was present. Applications of nitrate of silver rendered it worse. Then Dr. Clark's method was employed. This, at first, was painful. Four applications at intervals of three days were made, and now there is complete cure. I afterwards relieved the hernia, which was as large as a peck measure, by operation.

I wish it distinctly understood that I claim nothing original in this work. Dr. Kelly should be credited with a large part of the recent advances. Knowing that I was working along the same lines, he has urged me to report my results, which heretofore I have not done, and which I hope to do more extensively at no distant day.

### DISCUSSION.

Dr. Jacob Michaux asked Dr. Long if he had found hyperæsthesia of the external parts in the case last described.

Dr. Long: Yes, and it has been dissiminated four or five months.

Dr. Michaux, continuing, said that, in these cases, he had not had uniformly good results. Those in which he had succeeded best were those in which he had cauterized liberally, according to the old methods. In conditions of hyperæsthesia of the nymphæ and meatus, he had been much discouraged. He thought, therefore, that Dr. Long's remarks were interesting and valuable, and that the new method described had opened up a new field.

He described the following case as illustrative of failure: Woman, aged 36 years, primipara. There was intense hyperæsthesia of the mouth of the urethra; the desire to urinate was constant. The urethra being dilated, it was found to be inflamed, and was cauterized and washed out; the condition was thought to have been cured. The patient, who lived away from town, went home and soon reported herself as in the same condition. She returned and some little growths, curuncles, were removed from the meatus, producing no apparent benefit. Thinking, then, the trouble was due to the state of the nerves, a section of the meatus was cut out; but neither did this result in relief, nor did a second, deeper operation.

During all this period, extending over several years, suffering was intense, all treatment failing. Dr. Michaux thought it a case of cystitis, with a peculiar condition of the nerve fibres. It was one of the cases troubling him, and he would like to hear some discussion of it, with a view of eliciting instruction.

Dr. Hugh M. Taylor said that one feature should be added to Dr. Long's paper, and that was that the suggestion as to the safety of the ureters during laparatomy was made by a Virginian, Dr. Alexander Irvin, of Evington, Va. He suggested catheterization of the ureters.

Dr. Taylor said he had not yet had an oppoıtunity to test the efficiency of the instruments described, but had no doubt that, by their means, treatment and cure would be greatly facilitated.

Dr. J. N. Upshur remarked that, in the local treatment of any disease, we should always have one caution. The subject presented by Dr. Long was very interesting, but many diseases of organs treated locally are not primarily due to the organ itself. This is especially true of the bladder. Cystitis is often reflex, as from abrasion of the os uteri. In one case he saw no indication pointing to disease of the bladder itself. When the treatment was withdrawn from the organ it was cured. Retro-displacements, anteflexions, anteversions, hemorrhoids, anal fissures, nervous conditions, all give rise to irritation of the bladder; treatment, then, directed to that organ failing to relieve.

A case in point was as follows: Woman, with an intensely irritable bladder and frequent desire to void urine without satisfaction. As a matter of curiosity Dr. Upshur asked the patient to keep an account of the number of times she was up during one night, and was told that, between bed-time and 7:30 a. m, it was seventy times.

The woman was supposed to have been addicted to masturbation, and accused of it by former physicians, but vehemently denied it, and he was inclined to believe her. There was intense hyperæsthesia of the external genitals, and specular examination revealed a stenosed cervix, with marked anteflexion. Being before the days of curettage, he dilated without this procedure; but there was no benefit except a relief of dysmenorrhœa. He could find no other trouble except a tender spot in the lumbar spine, and advised her to seek another physician, which she did, but again received no benefit. Despairing, she gave up all treatment, and in the course of time her trouble disappeared. The doctor said he did not believe any bladder conditions existed, because none of them would have got well spontaneously. He thought it a case of hysteria based upon some nervous condition. He used this case as illustrative of the remark he made in the outset, that with the development of measures for local treatment we must not forget causes in other directions. Various deranged conditions of the stomach may cause vesical conditions, and a fairly common cause is, women do not drink enough water.

LAPAROTOMY—RECOVERY—WHITE DISCHARGE—WHAT WAS THE DISEASE?

Dr. W. T. Oppenhimer described a case, upon which he asked for information. Railroad man was suddenly attacked by violent pain in the liver, which he thought was due to a gall-stone. The patient passed a bad night, and by morning the pain had radiated over the entire abdomen and was accompanied by nausea and vomiting; in consequence the diagnosis was changed to appendicitis. Dr. I. H. White was called in consultation. The pain, in the meantime, being confined to the liver, gall-stone was the diagnosis agreed upon. The patient grew much worse; temperature fell, pulse became rapid and feeble, and tympanitis developed. In a second consultation Dr. White advised no operation. Dr. Geo. Ben Johnson, on seeing the case, said it was possibly appendicitis, but he, too, considering the man's condition, thought operation unjustifiable.

Dr. Oppenhimer told the patient his chances were almost hopeless; that an operation at least could do no more harm than hasten the end, and obtained his consent. At 12 p. m. he was removed to the Retreat for the Sick and the abdominal cavity opened. A fluid, chyliferous in appearance, flowed out. Everything, so far as ascertainable, appearing normal, the gut not sloughing, etc., the incision was closed and a gauze drain inserted. The following morning the swelling had abated, the temperature had fallen, and the discharge, which was profuse, became yellow. After four weeks the latter had become inspissated and could be squeezed out; at times being so firm that it had to be lifted out with forceps. The man recovered, and is now at work.

In answer to various questions, Dr. Oppenhimer stated that Dr. Hodge, on examining the discharge, found it gave the reaction of bile. The gall-bladder could not be reached. There was no jaundice. The man had had slight attacks of pain before, but they passed off in an hour or two.

